# Reproducing the Solvatochromism of Merocyanines by PCM Calculations

**DOI:** 10.3390/molecules29174103

**Published:** 2024-08-29

**Authors:** Andrés Aracena, Marcos Caroli Rezende, Sebastián Pizarro

**Affiliations:** 1 Instituto de Ciencias Naturales, Facultad de Medicina Veterinaria y Agronomía, Universidad de Las Américas, Sede Santiago, Campus La Florida, Avenida Walker Martínez 1360, La Florida, Santiago 8240000, Chile; 2 Facultad de Química y Biología, Universidad de Santiago de Chile, Estación Central, Santiago 9160000, Chile; marcos.caroli@usach.cl; 3 Escuela de Ingeniería Química, Pontificia Universidad Católica de Valparaíso, Avenida Brasil 2162, Valparaíso 2340000, Chile; sebastian.pizarro.a@mail.pucv.cl

**Keywords:** merocyanines, types of solvatochromism, solvent effects, PCM calculations, Fukui electrophilic function

## Abstract

Polarizable continuum methods (PCM) have been widely employed for simulating solvent effects, in spite of the fact that they either ignore specific interactions in solution or only partially reproduce non-specific contributions. Examples of three solvatochromic dyes with a negative, a positive and a reverse behavior illustrate the achievements and shortcomings of PCM calculations and the causes for their variable success. Even when qualitatively mimicking non-specific solvent effects, departures of calculated values from experimental data may be significant (20–30%). In addition, they can utterly fail to reproduce an inverted behavior that is caused by significant specific contributions by the solvent. As shown through a theoretical model that rationalizes and predicts the solvatochromism of phenolate merocyanines based on DFT (Density Functional Theory) descriptors in the gas phase, PCM shortcomings are to be held responsible for its eventual failure to reproduce experimental data in solution.

## 1. Introduction

Polarizable continuum models (PCM) constitute a rather crude, but nevertheless useful approach [1], to mimic the solvatochromic behavior of dyes in solution. As a simple and inexpensive approach employing a few parameters to characterize a solvent, they allow an overview of the solvatochromic response of a dye in different media, being frequently used to rationalize experimental observations [2,3,4,5,6,7,8,9,10,11,12,13]. Attempts to relate the experimentally observed solvatochromism of a family of dyes with particular structural features have utilized PCM calculations at various levels of theory. In many instances, their experimental behavior has been satisfactorily reproduced by theoretical calculations employing continuum models [6,10,11]. In other cases, however, PCM calculations have failed to reproduce experimental solvatochromic trends [3,5,9].

Phenolate betaines are a conspicuous example of solvatochromic compounds, where a phenolate donor group is conjugated with aryl or heteroaryl electron-accepting fragments. They have been the subject of different studies employing continuum models at various levels of theory [2,7,13,14,15,16]. Their solvatochromism is classified as negative, positive or reverse. A negative behavior is characterized by a hypsochromic shift of the solvent-dependent absorption band of the dye with the increased medium polarity, a positive behavior by a bathochromic shift, and a reverse or inverted solvatochromism by the change from a positive to a negative behavior at some polarity value [17]. Attempts to reproduce the solvatochromism of phenolate betaines with continuum models have had limited success [14,15]. This is often due to the inability of continuum models to mimic specific solvent effects, such as hydrogen bond interactions, responsible for the basicity (*SB*) and the acidity (*SA*) of a solvent [18]. By contrast, non-specific effects, such as the dipolarity (*SdP*) and the polarizability (*SP*) [18] of a solvent should be more adequately reproduced by PCM calculations. In fact, a recent publication has confirmed this expectation, showing that, irrespective of the employed level of calculations, solvatochromic trends are reproduced reasonably well with dyes that are the basis of polarizability and dipolarity scales [19]. An important consequence of these observations is that a proper knowledge of the major effects that contribute to the solvatochromism of a given dye can a priori decide whether PCM calculations will be successful in the reproduction of its behavior. Compounds whose solvatochromic response is mainly determined by non-specific solvent effects should have their behavior adequately reproduced by PCM calculations. Specific effects do not invalidate PCM calculations unless they significantly oppose non-specific effects, in which case theoretical results can lead to completely erroneous predictions. This is the case of many reverse dyes, with opposite contributions of the medium polarizability/dipolarity and acidity/basicity to their solvatochromism.

**Figure 1:**
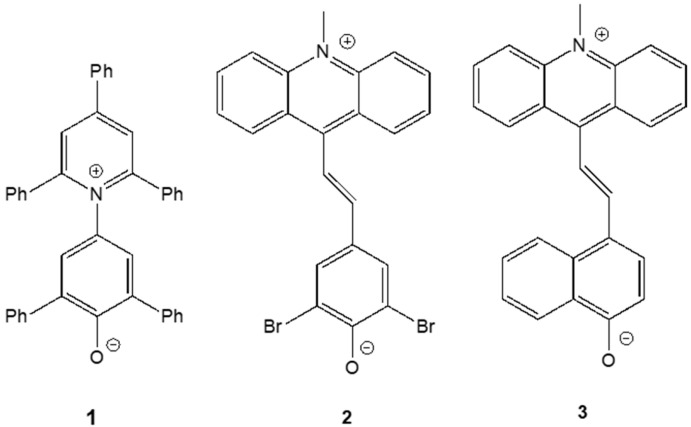
Structures of merocyanine dyes exhibiting a negative (**1**) [14,15,16], reverse (**2**) [20] and positive (**3**) [21] solvatochromism.

In the present work, we tested these predictions with three phenolate betaines, with a negative, a reverse and a positive behavior (Figure 1).

Compound **1**, Reichardt’s betaine, has been a subject of permanent interest [14,15,16] because of its various applications as an indicator and basis of the *E*_T_(30) polarity scale [22,23,24,25]. It is a classic example of a dye that exhibits negative solvatochromism. Compound **2** has been described by El Seoud et al. as a merocyanine that unquestionably exhibits a reverse behavior [20]. Finally, compound **3** is a classic example of another merocyanine showing positive solvatochromism [21].

Comparison of their experimental behavior with the theoretical solvatochromism resulting from PCM calculations illustrates the achievements and shortcomings of the continuum approach. As it still constitutes an inexpensive alternative for the simulation of solvent effects, employed for testing theoretical models and predictions of solvatochromic behavior in various solvents, it must be held responsible for their occasional failure to validate these models. This is shown in the second part of this communication, where the theoretical predictions based on structural considerations of a family of phenolate betaines are compared with the results obtained with PCM calculations for compounds **1**–**3**.

## 2. Results

### 2.1. Calculations of Betaines **1**–**3**

Structures were initially optimized in the gas phase and then in each solvent, employing the default PCM, using the integral equation formalism variant, and/or Truhlar’s SMD (Solvation Model based on Density) variation [26] at the B3LYP/6-311G(d) level of theory. Transition energies were then calculated with time-dependent density-functional theory (TD-DFT) single-point calculations, at the same level of theory.

The obtained theoretical S_0_→S_1_ transition energies *E*_T_, in kcal·mol^−1^, are listed in Table 1.

**Figure 2:**
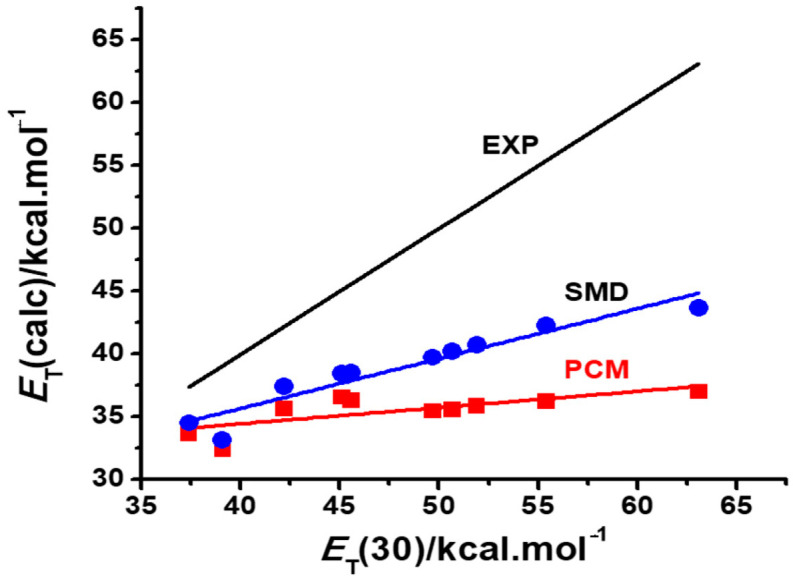
Plots of the calculated transition energies of dye **1**, *E*_T_(calc.) against the solvent *E*_T_(30) values, employing the standard PCM and the SMD option. Calculated values for both options are compared with the experimental straight line.

An initial evaluation of the standard PCM vs. the SMD option was carried out with Reichardt’s dye **1**. Plots of the two methods against the corresponding *E*_T_(30) values are reproduced in Figure 2.

Both methods reproduced qualitatively the negative solvatochromism of Reichardt’s betaine, although the SMD option proved superior to the standard PCM, as being more sensitive to its solvatochromic behavior in protic solvents, where non-specific hydrogen bond interactions play a major role. Although the SMD option does not take into account any specific solvent effects, being a variation of a continuum model, the corrections introduced in the values of the radii of some atoms lead to a better agreement with experimental results in protic solvents, where HBD contributions are important [3]. For this reason, we limited our calculations with merocyanines **2** and **3** to TD-DFT methods employing the SMD option.

**Figure 3:**
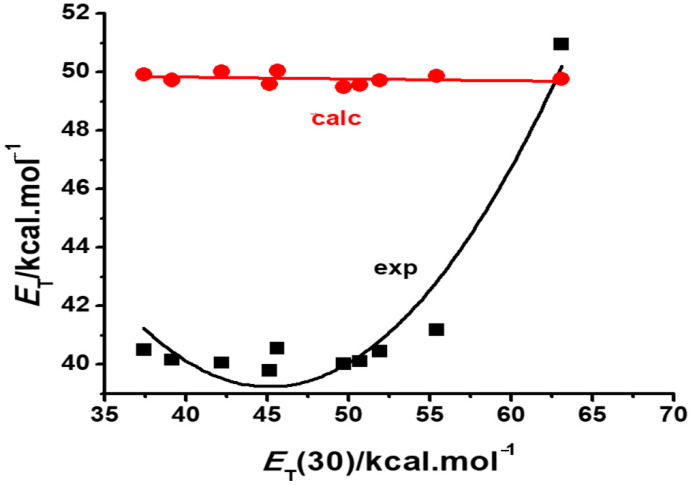
Comparison of the experimental (black squares) [20] and calculated (red circles) solvatochromic plots of dye **2** in various solvents.

In contrast with the reasonable qualitative reproduction of the negative solvatochromism of Reichardt’s betaine by continuum models, Figure 3 shows that PCM utterly fails to reproduce the inverted behavior of dye **2**, predicting a practically constant spectral response of the dye solutions to the medium polarity.

**Figure 4:**
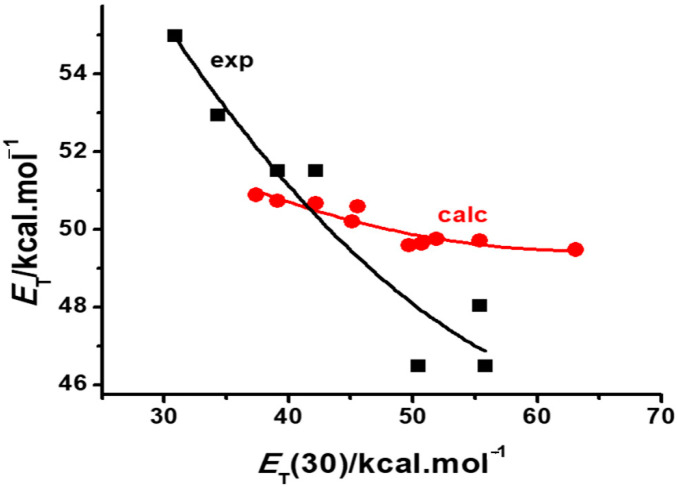
Comparison of the experimental (black squares) [21] and calculated (red circles) solvatochromic plots of dye **3** in various solvents.

A better performance is shown by the continuum model, when trying to reproduce the positive solvatochromic behavior of dye **3**, as can be seen in Figure 4. Although far from showing the same sensitivity to the medium polarity exhibited by the dye, the model correctly predicts a positive behavior for this solvatochromic betaine.

The above results can be rationalized by a comparison of the sensitivity of the dyes to specific and non-specific solvent effects. Linear regressions of the experimental solvatochromic transition energies for each dye, as a function of the non-specific medium polarizability (*SP*) and dipolarity (*SdP*), and specific acidity (*SA*) and basicity (*SB*), yielded Equations (1)–(3) for the corresponding betaines. For the sake of comparison, data from the same solvents were employed in the regression analyses of compounds **1** and **2** [20,24]. Equation (3) was obtained from reported values [21].

*E*_T_(**1**) = 31.13 + 10.39 *SdP* + 21.37 *SA* + 3.98 *SB* N = 27, r^2^ = 0.96 (1)

*E*_T_(**2**) = 47.74 − 6.53 *SdP* + 6.23 *SA* − 5.57 *SB* N = 27, r^2^ = 0.60 (2)

*E*_T_(**3**) = 56.51 − 6.94 *SP* − 1.74 *SdP* − 6.45 *SA* − 4.82 *SB* N = 7, r^2^ = 0.79 (3)

Absolute values for the coefficients of Equations (2) and (3) should be regarded with some caution, as the corresponding correlation coefficients are only reasonable. Nevertheless, more than their absolute values, the relative signs of specific and non-specific contributions are significant for our analysis. 

The negative solvatochromism of Reichardt’s dye **1** is known to be determined by the medium dipolarity (*SdP*) and acidity (*SA*). Although the contribution of this latter, specific solvent effect is more important than the former, both enhance the solvatochromic transition energy of the dye, leading to a negative behavior as polarity increases. The polarizable continuum model ignores specific contributions, being capable of reproducing to some extent the medium dipolarity only [19]. As a result, it still achieves a reasonable qualitative reproduction of the observed solvatochromism, as shown in Figure 2.

The results of the multilinear regression analysis of compound **3** (Equation (3)) show that all solvent effects reduce the solvatochromic transition energy of the dye, leading to a positive behavior with the increased medium polarity. Although the continuum model ignores the specific effects, like medium acidity and basicity, their contributions do not mask the non-specific effects of polarizability (*SP*) and dipolarity (*SdP*), which are taken into account, albeit to a smaller extent, by the PCM calculations [19]. As a result, also here, the continuum model achieves a reasonable qualitative reproduction of the observed solvatochromism, though much less sensitive to the increased polarity of the medium, as shown in Figure 4.

The total failure of calculations with the SMD option to reproduce the inverted solvatochromism of dye **2** (Figure 3) can be readily understood by inspection of Equation (2). The specific positive contribution by the medium acidity counterbalances the negative effect of the medium dipolarity, but this is not registered by the continuum model. As a result, PCM calculations predict a small decrease of the solvatochromic transition energy, even in the region where the solvent acidity becomes a major contributor to the observed increase of the *E*_T_ values.

### 2.2. Consequences of Continuum Model Limitations

Several attempts are found in the literature to rationalize the solvatochromic behavior of dyes based on theoretical calculations [2,3,14]. Efforts also are found, based on theoretical descriptors, to predict in silico the behavior of solvatochromic dyes in various solvents. By a proper choice of these descriptors and of a method to calculate them, it should be possible, for example, to predict the behavior of dyes **1**–**3**, discussed in the preceding section. Furthermore, in the case of the inverted behavior of dye **2**, a conceptually correct model should be capable not only of predicting its behavior, but also of estimating at which value of solvent polarity the expected inversion should take place. Unfortunately, this is often not true, if the calculation of theoretical descriptors in solution relies on continuum model approximations.

As an example, the behavior of betaines **1**–**3** will be analyzed in terms of a general model for the solvatochromism of merocyanines [27]. For a pair of conjugated donor/acceptor fragments exhibiting an internal charge transfer, the model postulates a gradual change from a positive to an inverted and to a negative behavior as the fragments or the solvating medium are changed. Such changes are reflected in theoretical descriptors derived from the density functional theory, such as fragment electrophilicities [28,29]. Thus, it should be possible, by calculations of DFT descriptors for a family of related dyes in the gas phase, to arrive at predictions of their solvatochromic behavior in solution. In addition, by the correct calculation of values for these descriptors in solution, it should be possible to reproduce the behavior of the dye and thus to validate the model.

**Figure 5:**
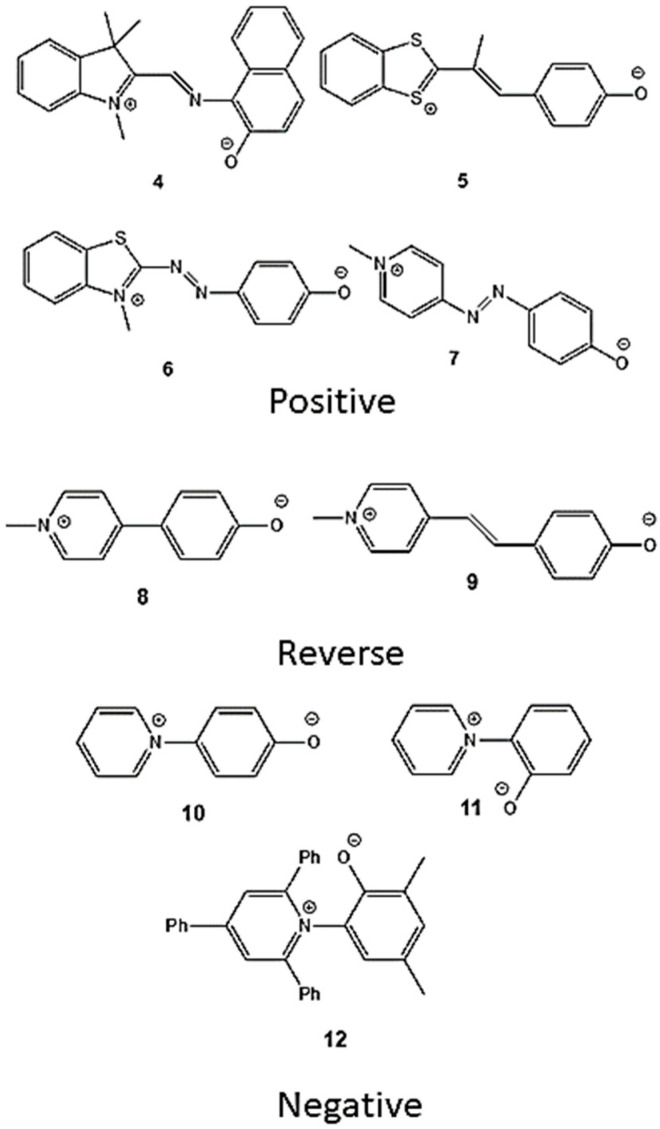
Examples of solvatochromic phenolate betaines exhibiting a positive [30,31,32], reverse [33,34] or negative [35,36] behavior.

As an illustration, the model can be applied to nine different phenolate betaines, showing a negative, a reverse or a positive behavior. Their structures, having in common a phenolate group conjugated with a heterocyclic moiety, are shown in Figure 5. To this set, betaines **1**–**3** were added, as a test for the prediction of their solvatochromism.

For a solvatochromic dye with a general structure A–B, its solvent-dependent band in a solvent arises from an internal charge transfer from A to B. The correct determination of the electrophilicities of its fragments A and B defines the amount and direction of the electronic flow in that solvent.

The electrophilicities of the two conjugated fragments are proportional to the group Fukui electrophilic function for the molecular fragment (*f*_G_^+^) obtained by summation of the condensed-to-atom Fukui functions *f*_k_^+^ of all atoms belonging to group G [28,29].

Values for these group Fukui functions are given in Table 2 for dyes **1**–**12**. The corresponding differences Δ*f*_G_^+^ = *f*_G_^+^ (heterocyclic) − *f*_G_^+^ (phenolate) are also given. They are an indication of the nature of the expected solvatochromism. Large positive values correspond to a truly negative solvatochromic behavior, where transition energies increase with the solvent polarity, measured with Reichardt’s *E*_T_(30) scale. Large negative values correspond to a truly positive solvatochromic behavior, where the opposite dependence of transition energies on solvent polarities is observed. Finally, small values, either negative or positive, define an inverted behavior.

**Figure 6:**
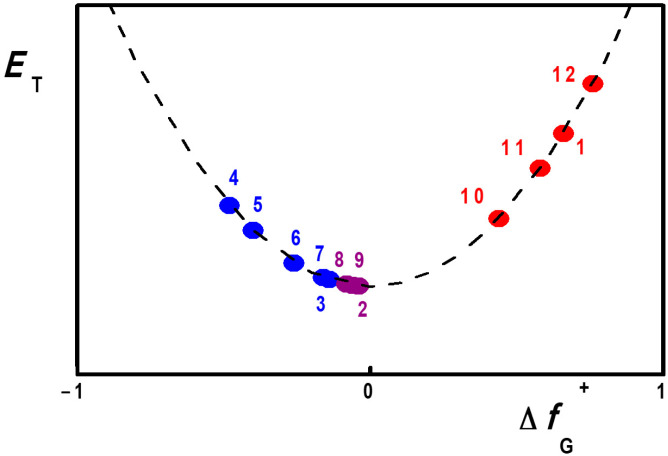
Schematic representation of the solvatochromic behavior of phenolate betaines **1**–**12** as a function of the difference Δ*f*_G_^+^ between the group Fukui electrophilic functions of their charge-transferring fragments A–B. Solvatochromic transition energies *E*_T_ vary with the relative electrophilicities of the two conjugated fragments, expressed in terms of their Fukui electrophilic functions. Dyes exhibiting negative solvatochromism (**1**,**10**–**12**) are shown in red, those exhibiting a positive behavior (**3**–**7**) are depicted in blue, those with an inverted behavior (**2**,**8**,**9**) in purple.

Figure 6 is a schematic representation of this general model [27] where the predicted behavior of a dye is situated along the abscissa expressing its difference in group Fukui functions Δ*f*_G_^+^. Notice that, in agreement with the experiment, positively solvatochromic dyes have a relatively large negative Δ*f*_G_^+^ value. As this value approaches zero, so does the dye assume a borderline positive, and then a reverse, behavior which evolves to a borderline negative and then a truly negative behavior, as the Δ*f*_G_^+^ value becomes larger and positive. Notice also that dyes **1**, **2** and **3** are also theoretically predicted to be examples of a truly negative, reverse and borderline positive behavior, respectively, in agreement with the experimental plots of Figure 2, Figure 3 and Figure 4.

Such predictions are made with gas phase calculations only. Their confirmation would require correct estimates of the group Fukui electrophilic functions in solution. However, if this is done with a PCM calculation, the results, as shown in Figure 2, Figure 3 and Figure 4, are only qualitatively reasonable, in the case of dyes **1** or **3**, or obviously false, in the case of the inverted behavior of **2**. Such a failure is not due to the theoretical postulates of the model but to the poor simulation of solvent effects on the dye electrophilicities, resulting from PCM or SMD calculations. As a consequence of continuum model limitations, theoretical models that purport to predict in silico the behavior of a solvatochromic merocyanine employing PCM to simulate solvent effects cannot be validated by calculations. Such a failure is not due to the fact that they are conceptually wrong, or that the choice of the theoretical descriptor in not adequate, but because PCM calculations fail to yield proper values for them in solution.

## 3. Materials and Methods

Calculations employed the Gaussian 16 package [37]. For the calculation of the solvatochromic transition energies of dyes **1**–**3**, all molecules had their structures initially optimized in the gas phase with the DFT (Density Functional Theory) method using the B3LYP functional and the 6-311G(d) basis set. Their structures were then optimized in solution with the aid of the PCM or SMD option, employing the same method of calculation. Their solvatochromic transition energies were obtained by single-point TD-DFT calculations employing the PCM or SMD over the optimized structures with their respective continuum model.

Fukui electrophilic functions were calculated by the method of Yang and Mortier [38] as the difference between the charges of an atom in the molecule, with N and N + 1 electrons. Summation of the function values thus obtained over all atoms of a group yielded the corresponding Fukui function *f*_G_^+^ of group G [28,29]. For the sake of consistency, calculations of structures **1**–**3**, employed the same method (B3LYP/6-31G(d)) utilized previously for the set of betaines **4**–**12**.

## 4. Conclusions

Continuum models are not capable of reproducing specific solvent effects derived, for example, from hydrogen bond interactions in solution. They are more effective in reproducing non-specific solvent effects, like the medium polarizability and dipolarity, although the reproduced trends describe a diminished sensitivity to the medium when compared with experimental data from actual polarizability and dipolarity sensors [19]. Such limitations must be taken into account when trying to reproduce the solvatochromism of new dyes with theoretical continuum models.

In the present communication, the limitations of the PCM model in the description and prediction of the solvatochromism of phenolate betaines were illustrated by comparing three dyes that exhibit a negative, a positive and an inverted behavior. Though ignoring specific solvent effects, continuum models provide qualitatively reasonable results when contributions from those effects are not significant or simply add to the contributions from non-specific (polarizability and dipolarity) solvent effects. However, the model utterly fails to reproduce or predict the behavior of phenolate betaines for which specific solvent contributions are significant and oppose those derived from non-specific effects.

As a corollary to the above considerations, attempts to predict and reproduce the solvatochromic behavior of phenolate betaines by theoretical tools derived from the density functional theory (DFT) can prove only partially successful, or even fail, if the employed DFT descriptors are calculated with the aid of continuum model approximations. Examples of this are dyes **1**, **2** and **3** correctly predicted to exhibit negative, inverted and positive solvatochromism, respectively, according to a general theoretical model for merocyanines and calculations in the gas phase [27]. However, calculations of their behavior in solution, applying the same model, can yield only a reasonable, qualitative agreement with experiment (dyes **1** or **3**), or even fail completely, in the case of the inverted behavior of dye **2**, because of the limitations of continuum models to estimate values for DFT descriptors in solution.

## Figures and Tables

**Table 1:** Calculated transition energies *E*_T_ in kcal·mol^−1^ in various solvents, employing the PCM or SMD and TD-DFT/6-311G(d) calculations.

Solvent	Solvent *E*_T_(30) Value/kcal·mol^−1^	Dye 1		Dye 2 ^a^	Dye 3 ^a^
		PCM	SMD	SMD	SMD
Cyclohexane	30.9	-	-	-	(54.98)
Benzene	34.3	-	-	-	(52.94)
Tetrahydrofuran	37.4	33.66	34.51	49.93 (40.50)	50.89
Chloroform	39.1	32.42	33.13	49.73 (40.16)	50.73 (51.51)
Acetone	42.2	35.63	37.41	50.02 (40.06)	50.68 (51.51)
Dimethylsulfoxide	45.1	36.57	38.42	49.60 (39.78)	50.21
Acetonitrile	45.6	36.30	38.51	50.05 (40.54)	50.60
1-Butanol	49.7	35.46	39.73	49.48 (40.02)	49.60
Benzyl alcohol	50.4	-	-	-	(46.49)
1-Propanol	50.7	35.60	40.24	49.56 (40.11)	49.63
Ethanol	51.9	35.88	40.74	49.72 (40.44)	49.75
Methanol	55.4	36.23	42.27	49.86 (41.18)	49.71 (46.49)
Formamide	55.8	-	-	-	(46.49)
Water	63.1	37.00	43.64	49.78 (50.96)	49.48

^a^ Experimental values in parentheses, for dyes **2** [20] and **3 [21]**.

**Table 2:** Values of group Fukui electrophilic functions for dyes **1**–**12**, calculated in the gas phase with the B3LYP/6-31G(d) method.

Compound	*f*_G_^+^ (Phenolate)	*f*_G_^+^ (Heterocyclic)	Δ*f*_G_^+^
**1**	0.17	0.83	0.66
**2**	0.54	0.46	−0.08
**3**	0.57	0.43	−0.14
**4:00** ^a^	0.74	0.26	−0.48
**5:00** ^a^	0.7	0.3	−0.4
**6:00** ^a^	0.63	0.37	−0.26
**7:00** ^a^	0.58	0.42	−0.16
**8** ^b^	0.48	0.52	−0.04
**9** ^b^	0.53	0.47	−0.06
**10** ^a^	0.28	0.72	0.44
**11** ^a^	0.21	0.79	0.58
**12** ^a^	0.12	0.88	0.76

^a^ from reference [28]; ^b^ from reference [29].

## Data Availability

Data are contained within the article.
